# A Comparison between Patient- and Physician-Reported Late Radiation Toxicity in Long-Term Prostate Cancer Survivors

**DOI:** 10.3390/cancers14071670

**Published:** 2022-03-25

**Authors:** Anna C. Nuijens, Arlene L. Oei, Anne Bouhuijs, Nicolaas A. P. Franken, Coen R. N. Rasch, Lukas J. A. Stalpers

**Affiliations:** 1 Department of Radiation Oncology, Amsterdam UMC, University of Amsterdam, Meibergdreef, 1105 AZ Amsterdam, The Netherlands; a.c.nuijens@amsterdamumc.nl (A.C.N.); a.l.oei@amsterdamumc.nl (A.L.O.); annebouhuijs@hotmail.com (A.B.); n.a.franken@amsterdamumc.nl (N.A.P.F.); 2 Center for Experimental and Molecular Medicine (CEMM), Laboratory for Experimental Oncology and Radiobiology (LEXOR), Amsterdam UMC, University of Amsterdam, Cancer Center Amsterdam, Meibergdreef, 1105 AZ Amsterdam, The Netherlands; 3 Department of Radiation Oncology, Leiden University Medical Center, 2333 ZA Leiden, The Netherlands; c.r.n.rasch@lumc.nl

**Keywords:** prostate cancer, external beam radiotherapy, late radiation toxicity, patient-reported outcome measures, gamma-H2AX assay

## Abstract

**Simple Summary:**

Radiotherapy is widely used as treatment for localized prostate cancer. Due to a high incidence and a good survival after treatment, a large number of prostate cancer survivors are at risk of developing late radiation toxicity. Symptoms may significantly affect quality of life; therefore, the monitoring of toxicities and evaluating their impact are increasingly important matters. Toxicities have always been assessed by physicians, but there is a growing interest in the use of questionnaires to be completed by patients themselves, so-called patient-reported outcome measures. The aim of this study was to compare both outcomes in long-term prostate cancer survivors, and to determine which outcome correlates best with a biological predictor of late radiation toxicity. In symptomatic patients, we found a low agreement; patients assigned greater severity to symptoms than the trial physician assistant did. Neither outcome correlated with the biological predictor. Consideration of both perspectives seems warranted to provide the best care.

**Abstract:**

Patient-reported outcome measures (PROMs) are advocated for the monitoring of toxicity after radiotherapy. However, studies comparing physician- and patient-reported toxicity show low concordance. In this study, we compared physician- and patient-reported toxicity in long-term prostate cancer survivors after radiotherapy, and we determined the correlation with a presumable risk factor for late toxicity: γ-H2AX foci decay ratio (FDR). Patients formerly included in a prospective study were invited to participate in this new study, comprising one questionnaire and one call with a trial physician assistant. Concordance was calculated for seven symptoms. Gamma-H2AX FDRs were determined in ex vivo irradiated lymphocytes in a previous analysis. Associations between FDR and long-term prevalence of toxicity were assessed using univariable logistic regression analyses. The 101 participants had a median follow-up period of 9 years. Outcomes were discordant in 71% of symptomatic patients; in 21%, the physician-assessed toxicity (using CTCAE) was higher, and, in 50%, the patients reported higher toxicity. We did not find a correlation between presence of toxicity at long-term follow-up and FDR. In conclusion, patients assigned greater severity to symptoms than the trial physician assistant did. Consideration of both perspectives may be warranted to provide the best care.

## 1. Introduction

External beam radiotherapy (EBRT) is widely used as primary treatment for localized prostate cancer. Due to the high incidence and good oncological outcome after treatment, there is a growing number of long-term survivors [1,2,3,4]. Consequently, a large number of patients are at risk of developing late radiation toxicity. In fact, around 30% of patients develop moderate or severe late radiation toxicity, although reported toxicity rates vary widely [5,6,7,8,9,10,11]. Symptoms can persist for a long time, if not permanently, and may significantly affect quality of life (QoL) [12,13,14]. Therefore, the monitoring of toxicities and evaluating their impact on QoL are increasingly important matters.

The quantification and monitoring of late radiation toxicity have always been of interest to radiation oncologists [15,16]. A frequently used instrument for this purpose is the National Cancer Institute’s Common Terminology Criteria for Adverse Events (CTCAE) [17,18]. In recent years, there has been increasing interest in the use of patient-reported outcome measures (PROMs) for the monitoring of toxicity, both in clinical trials and in daily clinical practice [19,20,21,22,23,24,25]. PROMs are questionnaires that are completed by patients to measure their perceptions of their own functional status and wellbeing. Historically, PROMs were designed for assessing treatment effectiveness and health-related QoL in clinical trials [26]. For this purpose they have been well validated [27]. In today’s clinical practice, they may also be used to assist in diagnosing conditions, to evaluate treatment burden, and to aid in improving physician–patient communication [21,28].

Remarkably, studies comparing physician-assessed toxicity with patient-reported symptoms generally report a poor agreement; in comparison to patients, physicians report symptoms less frequently and of lower severity [19,20,29,30,31]. Clinical trials with physician- and patient-reported outcomes after radiotherapy for prostate cancer are usually longitudinal in nature, reporting toxicity and QoL results as a change from baseline [22,25,32,33,34]. Studies directly comparing physician- and patient-reported outcomes after prostate radiotherapy are lacking. It can be envisioned that the poor agreement found in other patient populations also applies to prostate cancer patients. If so, it would be interesting to know which outcome correlates best with more objective findings.

Recently, we found that a less efficient repair of DNA double-strand breaks (DSBs) in ex vivo irradiated lymphocytes, as quantified by the γ-H2AX foci decay ratio (FDR), is an independent risk factor for the development of CTCAE grade ≥ 2 late toxicity in prostate cancer patients treated with EBRT [10]. Determining a patient’s γ-H2AX FDR before treatment may be an interesting additional tool, apart from looking into dose-volume parameters, to predict the individual risk for radiation toxicity. In turn, accurate prediction would allow for personalized treatment and, consequently, might prevent the development of severe late side-effects.

For the present study, we reached out to the prostate cancer patients included in our previous study to obtain long-term toxicity data [10]. The first aim of the present study was to report and compare the results of physician-assessed toxicity with patient-reported symptoms. The second aim was to determine which of both outcomes correlates best with the γ-H2AX FDR: the objective parameter associated with late radiation toxicity that was identified in our previous study.

## 2. Materials and Methods

### 2.1. Patients

Data from a previous prospective study were used in the present study [35]. The previous study was carried out to explore associations between biological and clinical parameters and toxicity after EBRT for prostate cancer. With local ethics committee approval, 200 patients were accrued at the AMC (now part of Amsterdam UMC) between 2009 and 2013. Data from 198 of these patients were analyzed. Participants were patients with newly diagnosed and histologically proven prostate cancer, to be treated by EBRT with curative intent. All participants provided written and verbal consent. Before the start of treatment, approximately 45 mL of blood was drawn. Using Ficoll gradient separation, lymphocytes were isolated and subsequently stored in liquid nitrogen. Toxicity was scored at the first follow-up visit after treatment and every 6 months thereafter by the treating physician according to the CTCAE version 4 (CTCAEv4.0) [17]. Toxicity scores were rated relative to symptoms at baseline.

Data from 179 patients were used for a recent analysis concerning the relevance of genetic predisposition, especially compared with dose-volume factors, to the risk of late radiation toxicity [10]. All patients were treated with intensity-modulated radiation therapy (IMRT). The median follow-up time of these 179 patients was 31 months. Patients were excluded if they had EBRT combined with brachytherapy, or when they were lost to follow-up within the first 3 months after treatment.

For the present study, the survival status of these 179 patients was assessed using the population register. Survivors were sent a letter, including a questionnaire, to ask to participate in this new study. Following recommendation of the local ethics committee, a renewed informed consent form (ICF) was also provided. Nonresponders received one written reminder after 4 weeks. Upon arrival of the completed questionnaire and signed ICF, patients were called by a trial physician assistant to assess toxicity according to the CTCAEv4.0. Patients were excluded if they had serious cognitive impairment that would complicate the correct completion of the questionnaire.

### 2.2. Assessment of Bowel and Urinary Toxicity

The physician’s evaluation of toxicity was performed using the CTCAEv4.0, in a one-time telephone contact [17]. The following items were considered clinically relevant for prostate cancer survivors treated with EBRT: abdominal pain, diarrhea, rectal hemorrhage, constipation, fecal and/or urinary incontinence, gastrointestinal and/or urinary fistula, urinary tract pain, urinary frequency and urgency, hematuria, and urinary retention. The focus was to score toxicity experienced during the last month before the telephonic assessment (i.e., period prevalence). Additionally, patients were asked about events in the past 5 years. Taking currently and previously gathered (original follow-up) data into account, cumulative incidences could also be determined. We did not look into the patients’ files to assess toxicity that occurred in the time period between the original (‘active’) follow-up and the current assessment.

Patient-reported outcomes (PROs) were assessed with the Dutch version of the validated EORTC QLQ-C30 and the prostate cancer-specific PR25 [36,37,38]. We focused on a subset of 14 questions regarding bowel and urinary symptoms: questions 16–17 of the C30 and questions 31–37 and 39–43 of the PR25 (Table 1). All scores were reported using a four-point Likert scale ranging from ‘not at all’ (1) to ‘very much’ (4).

### 2.3. Data Harmonization

To enable a direct comparison, the scales of both scoring systems were harmonized for seven symptoms by taking the definitions of each point on each scale and classifying them as ‘no’, ‘mild’, ‘moderate’, or ‘severe’, in terms of toxicity (Table 2). Patients with questionnaire responses of moderate or severe urinary frequency or urgency were considered as one group, with moderate toxicity as the recorded response. We decided so because the CTCAEv4.0 uses a three-point scale for these items. Furthermore, urinary frequency and urgency were recorded under the same denominator, and the highest reported grade or questionnaire response was used. The seven symptoms were diarrhea (question [Q]17), fecal incontinence (Q41), rectal hemorrhage (Q42), constipation (Q16), urinary tract pain (Q37), urinary frequency/urgency (Q31–34), and urinary incontinence (Q36).

### 2.4. γ-H2AX Foci Assay 

The lymphocytes were retrieved from the liquid nitrogen. Once thawed, they were irradiated with 1 Gy γ-rays from a ^137^Cs source. Gamma-H2AX foci kinetics were quantified in unstimulated G(0) cells. The γ-H2AX FDR was calculated by the ratio of foci found at 30 min to the number of foci found at 24 h after irradiation. For details regarding immunohistochemistry, γ-H2AX foci scoring, and determination of the FDR threshold, we refer to previously published methods [35,39].

### 2.5. Statistical Analysis 

To describe the study population, and to explore whether the study population differed from surviving patients not participating in this evaluation, descriptive statistics were used. Depending on normality of the distribution, the unpaired *t*-test or Mann–Whitney U test was used to analyze numeric values. Categorical data were assessed using the χ^2^ test.

Physician- and patient-reported outcomes were evaluated by counting frequencies of symptoms. The worst recorded symptom level was analyzed for each outcome. These data did not undergo formal statistical testing.

To describe the level of agreement, we calculated the percentage of concordance for the seven symptoms. The physician-reported data regarding toxicity experienced during the last month before the telephonic assessment were used for this calculation. Missing questionnaire items were excluded from paired analysis of those symptoms. In addition to measuring concordance percentages, we also measured percentages of pairs that disagreed by one or more points. Furthermore, we investigated what happens to the concordance rates when patients without toxicity (according to both physician and patient) are excluded from the analysis. No formal statistical tests were performed with these data.

Lastly, univariable logistic regression was performed to investigate whether the γ-H2AX FDR was associated with the long-term (period) prevalence of toxicity (i.e., according to the physician and/or to the patients). The FDR was dichotomized as < or ≥3.41, this threshold was determined in a previous retrospective study and validated in a prospective study and a recent re-evaluation of this latter study [10,35,40]. Groups were formed according to the presence (or lack) of moderate or worse toxicity in the previous week (PROM) or month (CTCAE). The strength of the association between γ-H2AX FDR and toxicity was expressed using the odds ratio (OR). A two-sided *p*-value of less than 0.050 was considered statistically significant. Statistical analysis was performed using IBM SPSS Statistics for Windows, version 26.0 (IBM Corp. Released 2019. Armonk, NY, USA).

## 3. Results

### 3.1. Patients

On 26 October 2020, 133 of 179 patients of the previous analysis were alive. Of the 133 survivors, seven declined participation, 10 did not respond to the invitation, and four were unfit due to an impaired cognition. Of the remaining 112 patients, complete data were available for 101 patients. Their median follow-up time was 9 years. Androgen deprivation therapy was administered to 86%. The most frequently (*n* = 99) prescribed dose was 77 Gy in 35 fractions (EQD2 80 Gy); two patients received a dose of 70 Gy in 35 fractions (EQD2 70 Gy). Apart from the EQD2 (α/β = 3 Gy; *p* = 0.031), there were no significant differences between participating and nonparticipating patients (*p*-values not shown; Table 3).

### 3.2. Physician-Reported Toxicity

As published before, in the original population of 179 patients with a median follow-up time of 31 months, the cumulative rates for urinary and bowel grade ≥ 2 late toxicities were 46% and 17%, respectively [10]. For the subgroup of 101 participants in the current study, these figures were 46% and 13% during the first posttreatment years (‘active FU’ column in Table 4).

In the last month before telephonic assessment, zero of the 101 participants suffered from grade ≥ 3 symptoms (Table 4). Grade 2 toxicity was recorded in 39 patients. Of these patients, 29 and 15 patients suffered from urinary and bowel toxicities, respectively. Urinary frequency and/or urgency (*n* = 17), and urinary incontinence (*n* = 14) were the most reported grade 2 toxicities. Fistulas were not recorded at all. The majority of patients (61%) had no or low-grade symptoms.

When patients were asked about their symptoms in the past 5 years, the outcome was largely comparable with the symptoms experienced in the month before telephonic assessment. For two patients, this was not the case. Both had been admitted to the hospital because of gross hematuria due to radiation cystitis. After discharge, both were successfully treated with hyperbaric oxygen therapy.

Taking the toxicity data of all assessed timepoints into account, only eight patients never experienced any symptom during the entire follow-up (‘ever’ column). The cumulative incidence of grade 3 toxicity was 9%.

### 3.3. Patient-Reported Toxicity

Sixteen patients suffered severely from at least one urinary or bowel symptom, i.e., 16 patients chose ‘very much’ as an answer at least once (Table 4). Urgency was the most reported symptom (Q33 *n* = 7; Q35 *n* = 5), followed by nocturia (*n* = 6) and frequency (*n* = 5). Bowel symptoms were primarily caused by fecal incontinence. Of these 16 patients, two patients reported to be very much limited in their daily activities because of their complaints. Thirty-one patients chose ‘quite a bit’ as an answer at least once. The majority of patients experienced either mild (44%) or no symptoms (10%).

### 3.4. Agreement between Physician- and Patient-Reported Outcomes

When including all patients, the mean concordance rate in toxicity scores was 80% (data not shown). However, concordance dropped to 29% when patients without symptoms (according to both patient and physician assistant) were excluded (Table 5). The agreement was highest for urinary tract pain (80%; *n* = 5), whereas, for constipation, there was a complete lack of agreement (*n* = 20). Regarding the patients with discordant scores, 50% of the patients reported higher levels of toxicity than the physician did. In 3% of the cases, there was a difference of two degrees. In 21% of the cases, the physician reported higher toxicity, and, in 3% of the cases, the physician score was higher by two degrees than that reported by patients in the questionnaire.

### 3.5. Correlation with γ-H2AX FDR

An FDR < 3.41 showed no association with the presence of either physician- or patient-reported toxicity (i.e., relative to the seven symptoms considered) at about 9 years of follow-up. Specifically, the odds of experiencing moderate or worse toxicity at about 9 years of follow-up were similar for patients with an FDR < 3.41 versus patients with an FDR ≥ 3.41 (CTCAE: OR 1.08, *p* = 0.856; PROM: OR 1.14, *p* = 0.764). When CTCAE outcome data regarding hematuria and urinary retention were also taken into account (i.e., nine instead of seven symptoms are considered), the OR marginally increased from 1.08 to 1.15 (*p* = 0.764). This latter analysis could not be performed with PROM data, since questions regarding hematuria and urinary retention are not included in the questionnaire.

## 4. Discussion

In this study of prostate cancer survivors, we compared physician-assessed toxicity with patient-reported symptoms at a median of 9 years after EBRT. We found a poor agreement between PROMs and CTCAE, with patients reporting greater severity more often than the trial physician assistant. Specifically, CTCAE and questionnaire scores were discordant in 71% of symptomatic patients; in 21%, symptoms were scored as more severe by the trial physician assistant, while, in 50%, the symptoms were scored as more severe by the patients.

These results are in line with other studies wherein PROs were compared with physician-reported outcome after radiotherapy. However, comparative studies in prostate cancer patients were lacking until now. In the study of Brouwers et al., breast cancer patients reported higher rates of toxicity than clinicians did, 10 years after treatment [30]. The mean concordance rate was 60%, which is also low, yet considerably higher than that in our study. This is probably due to an approach wherein all reported outcomes were considered, whilst we specifically looked into concordance in symptomatic patients. In the absence of symptoms, it seems likely that patient and physician will tend to agree with each other’s judgment. In another study, the physicians scored grade ≥ 3 (bladder, bowel, or vaginal) toxicity in 10% of cervical cancer survivors at 5 year follow-up, whereas 58% of the survivors reported severe symptoms at a median of 36 months later [29].

The cumulative incidence of severe (CTCAE grade ≥ 3) toxicity in our patient group was 9%. In contrast to this, the period prevalence of severe toxicity about 9 years after EBRT was 0%; not a single patient experienced severe toxicity in the month before telephonic assessment, according to our trial physician assistant. This suggests that the prevalence of toxicity ultimately decreases with duration of follow-up. In line with this, Syndikus et al. found that the prevalence of moderate and severe toxicities after prostate cancer radiotherapy generally increased up to 3 years and then lessened [41]. On the one hand, this can be explained by the natural course of things; on the other hand, problems may have been solved by an intervention [42,43]. For instance, this intervention could have been some kind of surgery or, as in two patients of our study, hyperbaric oxygen therapy. In accordance with this, Vistad et al. found that 20% of 147 cervical cancer survivors developed grade ≥ 3 toxicity in at least one organ (i.e., bowel, bladder, vagina) within the first five posttreatment years. The prevalence of grade ≥ 3 toxicity at 5 year follow-up was 10%; in several of the women, the problems were solved by major surgery, including five colostomies and two bowel resections [29].

In contrast to the physician’s assessment, 16 patients responded with ‘very much’ to at least one question. Remarkably, only two of them also reported to be ‘very much’ limited by their symptoms in their daily activities. This discrepancy may be explained by the influence of several personal factors, such as coping style, comorbidity, and emotional disturbance. In contrast, CTCAE scoring is guided by concrete matters such as prescription of medication or hospital admission and, therefore, seems less easily affected by subjective factors.

For the present study, we reached out to former participants of a prospective study in which we found that impaired DNA DSB repair in ex vivo irradiated lymphocytes, as quantified by γ-H2AX FDR, was the most significant risk factor for the development of CTCAE grade ≥ 2 late urinary and bowel toxicity [10]. Currently, we unexpectedly did not find an association between toxicity grade and FDR. One explanation could be that we changed the endpoint from grade ≥ 2 toxicity at any time during follow-up (i.e., cumulative incidence) to grade ≥ 2 toxicity at long-term follow-up (i.e., prevalence). Our previously used endpoint may be more useful in this scenario, since prevalence numbers only reflect the situation (absence/presence of toxicity) at a specific moment or period in time and, hence, a lot of information is lost. For instance, a urethral stricture requiring surgical intervention will always end up in cumulative toxicity rates, whereas it will disappear in the prevalence numbers once treated successfully. If we repeated the analysis using the highest toxicity grade recorded ever during follow-up (i.e., cumulative incidence instead of period prevalence at about 9 years), we found an association between FDR and toxicity reaching statistical significance (*p* = 0.059; OR 2.32). Interestingly, if we only took the CTCAE data from the first posttreatment years of the current 101 participants into account (also cumulative incidence, but median follow-up about 31 months), then an FDR < 3.41 was identified as a significant risk factor (*p* = 0.034; OR 2.49). The loss of significance with increased duration of follow-up may be explained by increased obscuring of our primary outcome, due to symptoms that came with age and, thus, did not originate from radiation toxicity. 

This, in fact, is the main limitation of this study. Several symptoms now attributed to radiation toxicity could very well originate from other causes. For example, lower urinary tract symptoms are quite common in the elderly population [44]. This plausible presence of causes other than radiation toxicity complicates finding associations between toxicity and potential risk factors. Nevertheless, one can assume that the level of (dis)agreement between physician- and patient-reported outcomes is not affected by the cause of the symptoms. Thus, although we have to be cautious when interpreting the results with respect to the absolute toxicity levels, we consider the analyses of (dis)agreement levels reliable, particularly since all toxicity was scored by the same physician assistant, thereby avoiding inter-individual differences in the interpretation of symptoms. A second limitation may be the use of different time frames for the toxicity assessments, i.e., 1 week in the case of patient-reported toxicity and 1 month in the case of physician-reported toxicity. We experienced that it could easily take a week to actually get a patient on the phone at an appropriate moment to assess possible toxicities, counted from the day on which the questionnaire was filled in. For this reason, we decided to apply a different time frame for the physician-reported toxicity, to try to maintain an overlap in both time frames. Nevertheless, a minor impact on the results of the comparison cannot be ruled out. Ideally, both assessments would have been carried out on the same day; however, this was not logistically feasible.

Unfortunately, on the basis of the data provided by the current study, we cannot conclude which outcome measure correlates best with FDR. Despite this lack of clarity, for several reasons, it can be concluded that PROMs, at least the one used in this study, seem inappropriate tools for evaluating toxicity. Firstly, questions regarding relevant items, such as hematuria, are missing. Secondly, missing data (i.e., blank questions) and misinterpretation of questions seem to form a substantial problem with this specific PROM. Question 38 of the PR25 regarding incontinence aids is situated in the middle of the front page, and it only has to be answered when aids are used. We noticed several times that questions 39–46 on that same page were erroneously left blank. On the contrary, the separate section about sexual experiences (Q52–55), which only had to be answered when being sexually active in the past 4 weeks, was also filled in by five patients that were not sexually active (Q51). Moreover, as stated before, PROs are likely to be influenced by personal factors.

This does not mean that PRO(M)s are useless. It is this influence of personal factors that makes PROMs very useful tools for providing insight into the burden of toxicity. This was actually shown by the study of Basch et al., in which CTCAE assessments better predicted unfavorable clinical events, whereas patient reports (PRO-CTCAE) better reflected daily health status [20]. Due to the complementary nature of both physician and patient perspectives, consideration of PROs helps to gain a holistic view of patient status. Therefore, in daily practice, physicians would ideally be provided with PROs to counsel patients, encourage discussions, and guide decisions.

## 5. Conclusions

To the best of our knowledge, our study is the first to directly compare physician- and patient-reported symptoms in prostate cancer survivors. In the symptomatic group, we found a poor agreement between physician and patient at a median follow-up of 9 years after EBRT. Whether one or the other outcome correlates better with the γ-H2AX FDR, a marker for genetic predisposition that we established in a prior study, remains unclear. We may not need an answer to this question. There is ample reason to believe that PROMs and physician assessment provide complementary information and, thus, one should not be replaced by the other. Consideration of both perspectives may be warranted to provide the best care.

A new prospective study into risk factors of late radiation toxicity is currently in progress, now comprising both physician- and patient-reported outcomes. We hope to further validate the γ-H2AX FDR as a predictive marker within a population of prostate and cervical cancer patients, with the final aim of developing a reliable predictive model to support decision making in radiation therapy practice.

## Figures and Tables

**Table 1:** Subset of 14 questions of the EORTC QLQ-C30 and PR25 regarding genitourinary and gastrointestinal toxicity, including 10 questions enabling a direct comparison with physician-reported toxicity, as graded according to the CTCAE version 4.0.

Q	During the Past Week	Not at All	A Little	Quite a Bit	Very Much
EORTC QLQ-C30 (version 3)					
16	Have you been constipated?	1	2	3	4
17	Have you had diarrhea?	1	2	3	4
EORTC QLQ-PR25 (phase IV module)					
31	Have you had to urinate frequently during the day?	1	2	3	4
32	Have you had to urinate frequently at night?	1	2	3	4
33	When you felt the urge to pass urine, did you have to hurry to get to the toilet?	1	2	3	4
34	Was it difficult for you to get enough sleep, because you needed to get up frequently at night to urinate?	1	2	3	4
35	Have you had difficulty going out of the house because you needed to be close to a toilet?	1	2	3	4
36	Have you had any unintentional release (leakage) of urine?	1	2	3	4
37	Did you have pain when you urinated?	1	2	3	4
39	Have your daily activities been limited by your urinary problems?	1	2	3	4
40	Have your daily activities been limited by your bowel problems?	1	2	3	4
41	Have you had any unintentional release (leakage) of stools?	1	2	3	4
42	Have you had blood in your stools?	1	2	3	4
43	Did you have a bloated feeling in your abdomen?	1	2	3	4

Abbreviation: Q = question.

**Table 2:** Grade-response outcome pairs for concordant outcomes of no, mild, moderate, and severe toxicity, respectively.

Method	Toxicity			
	No	Mild	Moderate	Severe
CTCAE version 4.0—grade	0	1	2	3
QLQ-C30/PR25—response	1	2	3	4

**Table 3:** Characteristics of participating, nonparticipating, and deceased patients.

Characteristic	All Patients *n* = 179	Participants *n* = 101	Nonparticipants *n* = 32	Deceased *n* = 46
Median (IQR) FU, years	9 (8–10)	9 (9–10)	9 (9–10)	6 (4–8)
Mean (IQR) age, years *	69.2 (64–74)	68.2 (64–73)	69.8 (66–75)	71.2 (65.5–77)
Mean (IQR) KPS *	97.3 (100–100)	97.4 (100–100)	97.4 (100–100)	97.1 (92.5–100)
Mean (IQR) Gleason score *	7.4 (7.0–8.0)	7.3 (7–8)	7.5 (7–9)	7.4 (7–9)
Mean (IQR) PSA, ng/mL *	31.4 (8.9–27.3)	25.9 (8.8–27.0)	42.4 (7.7–33.7)	35.8 (10.8–28.9)
Mean (SD) FDR †	3.11 (0.70)	3.16 (0.70)	3.10 (0.67)	3.00 (0.71)
Highest late toxicity grade during FU of original study ‡				
0	47 (26)	28 (28)	12 (37)	7 (15)
1	31 (17)	18 (18)	5 (16)	8 (17)
2	84 (47)	48 (47)	10 (31)	26 (57)
≥3	17 (10)	7 (7)	5 (16)	5 (11)
T-stage				
T1	29 (16.2)	15 (14.9)	6 (18.8)	8 (17.4)
T2	74 (41.3)	48 (47.5)	10 (31.3)	16 (34.8)
T3	68 (38.0)	32 (31.7)	15 (46.9)	21 (45.7)
T4	8 (4.5)	6 (5.9)	1 (3.1)	1 (2.2)
EQD2, Gy §				
70	7 (4)	2 (2)	3 (9)	2 (4)
80	170 (95)	99 (98)	28 (88)	43 (94)
83	2 (1)	0 (0)	1 (3)	1 (2)
Androgen deprivation	154 (86)	87 (86)	28 (88)	39 (85)
TURP *	27 (15)	10 (10)	6 (19)	11 (24)
CVD *	118 (65.9)	71 (70.3)	20 (62.5)	27 (58.7)
Diabetes mellitus *	32 (18)	16 (16)	6 (19)	10 (22)
Smoking status *				
Current	30 (17)	13 (13)	7 (22)	10 (22)
Former	60 (33)	37 (37)	9 (28)	14 (30)
Never	89 (50)	51 (50)	16 (50)	22 (48)

Abbreviations: IQR = interquartile range; FU = follow-up; KPS = Karnofsky Performance Status; PSA = prostate-specific antigen; SD = standard deviation; FDR = foci decay ratio; EQD2 = equivalent dose in 2 Gy fractions; TURP = transurethral resection of the prostate; CVD = cardiovascular disease. * Before start of radiation therapy. ^†^ As determined in the original study. ^‡^ Late bowel and/or urinary toxicity, graded according to the CTCAE version 4.0. Late side-effects were defined as those appearing more than 3 months after the completion of radiotherapy. ^§^ We used an α/β ratio of 3 Gy. The group of nonparticipants consists of nonresponders, patients that declined participation, unfit patients, and patients with incomplete data. Data are presented as *n* (%) unless otherwise noted.

**Table 4:** Physician- and patient-reported toxicity in 101 prostate cancer patients treated with external beam radiotherapy between 2009 and 2013.

		Physician *				Patient ^†^
Highest Reported Toxicity		Active FU	Past 5 Years	Last Month	Ever	Last Week
No	Neither GU nor GI ^‡^	28	28	24	8	10
	GU ^#^	46	35	35	18	11
	GI ^#^	62	70	71	42	52
Mild	Either GU or GI ^§^	18	32	38	25	44
	GU ^¶^	9	32	37	25	47
	GI ^¶^	26	13	15	27	38
Moderate	Either GU or GI ^§^	48	39	39	59	31
	GU ^¶^	42	32	29	52	28
	GI ^¶^	10	18	15	29	7
Severe	Either GU or GI ^§^	7	2	0	9	16
	GU ^¶^	4	2	0	6	15
	GI ^¶^	3	0	0	3	4

Abbreviations: GU = genitourinary; GI = gastrointestinal; FU = follow-up. * Graded according to the CTCAE version 4.0. Items: abdominal pain, diarrhea, rectal hemorrhage, constipation, fecal and/or urinary incontinence, gastrointestinal and/or urinary fistula, urinary tract pain, urinary frequency and urgency, hematuria, and urinary retention. ^†^ Assessed with EORTC-QLQ-C30 and -PR25. Questions: 16–17 of C30 and 31–37, 39–43 of PR25. ^‡^ Patients without any symptom. ^#^ Patients without GU symptoms but could either have GI symptoms or no symptoms at all, and vice versa for patients without GI symptoms. ^§^ Patients with any mild/moderate/severe symptom, regardless of GU or GI nature. ^¶^ Patients with GU symptoms but could also have GI symptoms or no symptoms at all, and vice versa for patients with GI symptoms. Data are presented as the number of patients.

**Table 5:** Agreement of toxicity in symptomatic patients, as reported by physician and patient through telephonic assessment and completed questionnaire, respectively.

Symptom	*n* *	CTCAE < Q (2)	CTCAE < Q (1)	CTCAE = Q	CTCAE > Q (1)	CTCAE > Q (2)
Diarrhea	18	0 (0%)	11 (61%)	3 (17%)	4 (22%)	0 (0%)
Fecal incontinence	17	1 (6%)	11 (65%)	3 (17%)	2 (12%)	0 (0%)
Rectal hemorrhage	9	0 (0%)	5 (56%)	2 (22%)	2 (22%)	0 (0%)
Constipation	20	3 (15%)	8 (40%)	0 (0%)	6 (30%)	3 (15%)
Urinary tract pain	5	0 (0%)	1 (20%)	4 (80%)	0 (0%)	0 (0%)
Frequency/urgency	88	2 (2%)	51 (58%)	34 (39%)	1 (1%)	0 (0%)
Urinary incontinence	37	0 (0%)	10 (27%)	11 (30%)	14 (38%)	2 (5%)
Mean		3.3%	46.6%	29.3%	17.9%	2.9%

Abbreviations: CTCAE = Common Terminology Criteria for Adverse Events version 4.0; Q = questionnaire. * Number of patients that had the specific symptom, according to the physician and/or to themselves. CTCAE < Q means that, through telephonic assessment, lower grades of toxicity were scored by the physician than patients reported in the questionnaire, with (2) and (1) corresponding to a difference of two and one degrees, respectively, and vice versa in case of CTCAE > Q. CTCAE = Q means full agreement.

## Data Availability

Due to the sensitive nature of the questions asked in this study, survey respondents were assured that raw data would remain confidential and would not be shared.
